# Alzheimer's Disease Beyond Amyloid: Lessons From Atherosclerosis

**DOI:** 10.1002/acn3.70533

**Published:** 2026-09-16

**Authors:** Marcello Ciaccio, Luisa Agnello

**Affiliations:** ^1^ Department of Biomedicine, Neurosciences, and Advanced Diagnostics, Institute of Clinical Biochemistry, Clinical Molecular Medicine, and Clinical Laboratory Medicine University of Palermo Palermo Italy; ^2^ Department of Laboratory Medicine University Hospital Paolo Giaccone Palermo Italy

**Keywords:** Alzheimer's disease, biological Alzheimer's disease, biological resilience, clinical Alzheimer's disease, precision medicine

## 1 Introduction

Amyloid pathology has shaped every aspect of Alzheimer's disease research, from pathogenetic theories and biomarker development to the biological definition of the disease and the advent of disease‐modifying therapies [1, 2]. Yet increasing evidence indicates that, although amyloid is necessary for the biological definition of Alzheimer's disease, it is insufficient to explain the remarkable heterogeneity in clinical presentation and disease progression [3].

Sensitive fluid and imaging biomarkers have revealed that amyloid pathology begins decades before symptom onset and is frequently detected in cognitively unimpaired individuals [4]. Observational and neuropathological studies have consistently shown that individuals with comparable amyloid burdens may follow remarkably different clinical trajectories, ranging from lifelong cognitive resilience to rapidly progressive dementia [5]. Furthermore, the advent of anti‐amyloid therapies has reinforced the distinction between target engagement and clinical benefit, demonstrating that successful amyloid removal alone does not fully account for the complexity of disease progression [6]. Together, these findings challenge a deterministic interpretation of amyloid pathology and indicate that, although amyloid is essential for the biological definition of Alzheimer's disease, it is not sufficient to establish its clinical diagnosis. Thus, the biological and clinical definitions of Alzheimer's disease should be regarded as distinct, although related, concepts, underscoring that amyloid positivity alone does not equate to clinical Alzheimer's disease [7, 8].

This apparent paradox is not unique in medicine. Cardiovascular research has undergone a remarkably similar conceptual evolution. Atherosclerotic plaque was once regarded as the direct cause of myocardial infarction and stroke. Today, however, cardiovascular disease is understood as the consequence of a dynamic interplay among plaque biology, inflammation, endothelial dysfunction, thrombosis, metabolic health, genetics, environmental exposures, and lifestyle factors [9]. Accordingly, clinical risk is not determined simply by the presence or extent of plaque, but by the biological processes that govern plaque stability, vulnerability, and the individual's overall susceptibility to disease [10]. This conceptual shift has transformed cardiovascular medicine, moving the field from a lesion‐centered view of disease toward an integrated model of biological risk.

Alzheimer's disease has reached a comparable stage of conceptual maturity. Rather than considering amyloid deposition as a sufficient determinant of dementia, we propose that it should be viewed as the biological substrate upon which a broader network of pathological and protective mechanisms operates. Amyloid accumulation may initiate or facilitate downstream processes, but the transition from biological pathology to clinical disease is ultimately shaped by their interaction. Tau propagation, neuroinflammation, synaptic dysfunction, cerebrovascular injury, metabolic alterations, genetic susceptibility, co‐existing pathologies, and mechanisms of biological and cognitive resilience may amplify or mitigate the effects of amyloid pathology, thereby influencing whether, when, and how cognitive impairment emerges [11]. Within this framework, amyloid remains indispensable to the biological definition of Alzheimer's disease, but its clinical significance depends on the biological context in which it occurs. This multidimensional perspective may help explain why individuals with apparently comparable amyloid burden can follow markedly different clinical trajectories, ranging from prolonged cognitive stability to rapidly progressive dementia.

We therefore propose a conceptual shift that preserves the central role of amyloid in the biological definition of Alzheimer's disease while moving beyond an exclusively amyloid‐centric interpretation of its clinical expression and progression.

## 2 Lessons From Atherosclerosis: From Plaque‐Centered Disease to Biological Risk

The history of atherosclerosis provides a compelling example of how the interpretation of a pathological hallmark can evolve as biological knowledge advances. For decades, atherosclerotic plaque was regarded as the direct anatomical substrate of myocardial infarction and ischemic stroke [12]. Disease severity was largely equated with the extent of luminal stenosis, and the presence of plaque was considered the principal determinant of cardiovascular events. However, this view proved insufficient to explain the marked heterogeneity in clinical outcomes observed among individuals with apparently similar atherosclerotic burden [13].

A series of pathological, imaging, and clinical studies fundamentally changed this perspective. It became evident that many severe stenotic plaques remain clinically silent throughout life, whereas acute cardiovascular events frequently originate from lesions producing only modest luminal narrowing. The risk of myocardial infarction or stroke therefore depends not simply on the presence or size of the plaque, but on its biological behavior. Features such as inflammatory activity, lipid composition, fibrous cap integrity, neovascularization, intraplaque hemorrhage, endothelial dysfunction, and thrombotic susceptibility determine whether an atherosclerotic plaque remains stable or progresses to rupture and clinical disease [14, 15].

This conceptual evolution profoundly transformed cardiovascular medicine. Rather than focusing exclusively on detecting plaque, contemporary prevention strategies seek to identify the biological mechanisms that confer plaque vulnerability and to modify the systemic factors that influence disease progression. Cardiovascular risk is now understood as the result of a dynamic interaction between the pathological substrate and a wide range of modifiers, including age, genetics, hypertension, diabetes, dyslipidemia, obesity, smoking, chronic inflammation, physical inactivity, diet, and environmental exposures [16]. Consequently, the presence of atherosclerotic plaque is regarded as an indicator of increased biological risk rather than an inevitable predictor of myocardial infarction or stroke.

A comparable heterogeneity characterizes Alzheimer's disease and is supported by recent systematic evidence. A meta‐analysis of the prognosis of mild cognitive impairment showed substantial variability in clinical trajectories, with only a proportion of individuals progressing to dementia, while others remained cognitively stable or reverted to normal cognition [17]. Importantly, a meta‐analysis of biomarker‐defined cohorts showed that amyloid positivity was associated with an increased risk of clinical progression, but that this risk was markedly higher when concomitant tau pathology was present [18]. These findings indicate that progression along the Alzheimer's disease continuum is highly heterogeneous and that amyloid status alone provides only partial information about subsequent clinical trajectory. This heterogeneity closely parallels the variable clinical expression of atherosclerotic disease, in which the pathological lesion must be interpreted within the broader biological context that determines its clinical consequences.

**FIGURE 1:**
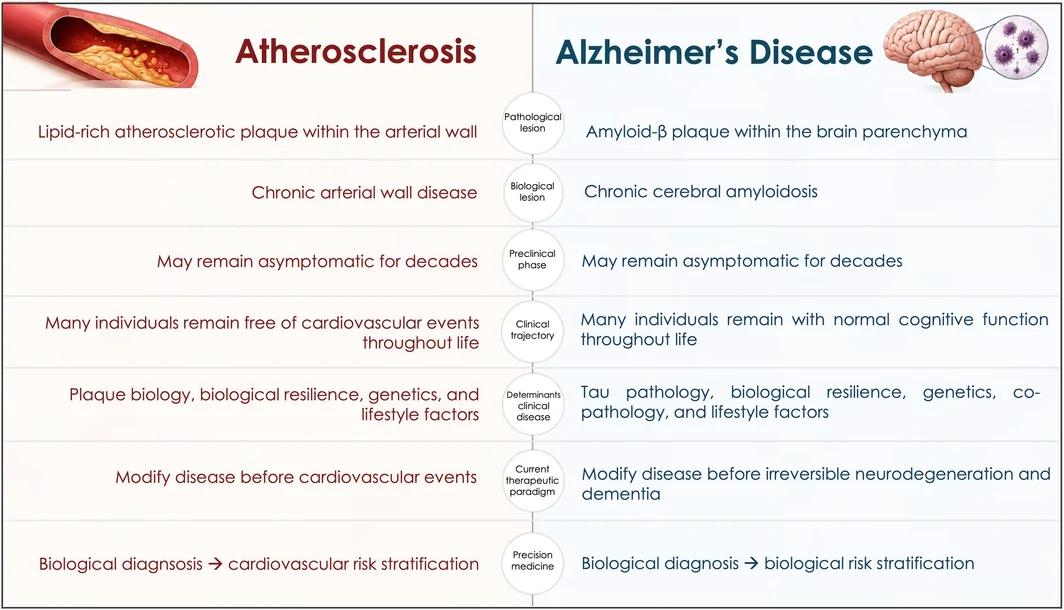
A shared conceptual framework for atherosclerosis and Alzheimer's disease. This figure illustrates the conceptual analogy between atherosclerosis and Alzheimer's disease, highlighting how both conditions evolve from the presence of a defining pathological lesion to a clinically heterogeneous disease shaped by multiple biological modifiers.

This analogy extends beyond pathology to disease prevention and precision medicine. The success of cardiovascular medicine has not resulted from eliminating atherosclerosis altogether, but from understanding the factors that influence its clinical expression and intervening before irreversible events occur. In Alzheimer's disease, tau propagation, neuroinflammation, cerebrovascular dysfunction, metabolic health, genetic susceptibility, and mechanisms of biological resilience may similarly represent determinants of disease progression and potential targets for precision prevention and treatment (Figure 1).

## 3 Converging Evidence for a New Biological Paradigm

Multiple and independent lines of evidence support this multidimensional interpretation of Alzheimer's disease. Particularly informative observations derive from genetically determined disease, longitudinal population‐based cohorts, therapeutic trials, and neuropathological studies of mixed pathologies.

The strongest direct evidence that amyloid accumulation is not sufficient to cause dementia comes, paradoxically, from the population in which the amyloid cascade hypothesis is best supported: carriers of autosomal dominant, fully penetrant AD mutations [19]. The Colombian PSEN1 E280A kindred from Antioquia, the largest such family in the world, typically develops mild cognitive impairment around age 44 and dementia around age 49, with amyloid deposition detectable more than two decades before symptom onset [20]. Within this same, genetically homogeneous cohort, two carriers have now been reported who accumulated amyloid burden comparable to, or exceeding, that of their symptomatic relatives yet remained free of dementia for decades beyond the expected age of onset [21, 22]. One carrier, homozygous for the rare APOE3 Christchurch variant, showed high amyloid‐PET signal but only limited tau spread and cortical atrophy, and did not develop dementia until her 70s, roughly three decades later than expected. A second carrier, bearing a rare variant in RELN (COLBOS), showed a similarly protected trajectory with delayed onset of several years despite a comparably high amyloid burden. In both cases the protective mechanism appears to act not on amyloid production or clearance but downstream, limiting tau propagation and the associated neuroinflammatory and neurodegenerative cascade. These cases provide compelling evidence that amyloid burden and clinical dementia can be uncoupled, and that the determinants of this uncoupling, that is, tau‐propagation control, glial reactivity, synaptic and vascular resilience, are precisely the downstream, non‐amyloid processes [23].

Importantly, this principle extends beyond rare genetic forms of the disease. Population‐based cohorts and biomarker studies have consistently shown that a substantial proportion of cognitively unimpaired older adults fulfill biological criteria for Alzheimer's disease while remaining clinically normal for prolonged periods [24, 25]. Although amyloid positivity increases the probability of future cognitive decline, progression is highly heterogeneous, with many individuals remaining cognitively stable for years or throughout life. Conversely, individuals with apparently similar amyloid burden frequently exhibit markedly different rates of tau accumulation, neurodegeneration, and cognitive decline [26]. These observations indicate that amyloid positivity identifies a state of increased biological susceptibility rather than an inevitable clinical destiny.

Evidence from therapeutic trials further supports this interpretation. The development of anti‐amyloid monoclonal antibodies has unequivocally demonstrated that cerebral amyloid can be substantially reduced in vivo [27]. Nevertheless, the associated clinical benefits have been consistently modest compared with the magnitude of amyloid removal [28]. Across pivotal Phase III trials of lecanemab and donanemab, treatment slowed cognitive decline but did not arrest disease progression, despite profound reductions in amyloid burden [29]. These findings should not be interpreted as a failure of the amyloid hypothesis; rather, they suggest that amyloid removal alone may be insufficient once downstream pathological cascades, including tau spread, synaptic dysfunction, neuroinflammation, and neuronal loss, have become established. In this respect, therapeutic experience mirrors biological observations: modifying the pathological substrate does not necessarily abolish the complex network of mechanisms ultimately responsible for cognitive decline.

A further and often underappreciated source of evidence comes from community‐based neuropathological studies. Unlike clinic‐based autopsy series, cohorts such as the Religious Orders Study (ROS), the Rush Memory and Aging Project (MAP), and the Medical Research Council Cognitive Function and Aging Study (MRC CFAS) examine participants irrespective of their ante‐mortem clinical diagnosis, providing a more representative picture of brain aging in the general population [30]. These studies have consistently demonstrated that pure Alzheimer's disease is the exception rather than the rule in older adults. Instead, most individuals with dementia harbor multiple co‐existing pathologies, including Alzheimer's neuropathologic change together with cerebrovascular disease, Lewy body pathology, limbic‐predominant age‐related TDP‐43 encephalopathy (LATE), hippocampal sclerosis, argyrophilic grain disease, and other age‐related proteinopathies [31]. The recognition of LATE as a distinct neurodegenerative entity has been particularly informative, as it frequently mimics Alzheimer's disease clinically while remaining neuropathologically, and often genetically, distinct [32]. These observations have profoundly changed our understanding of dementia in late life, revealing that cognitive decline most commonly reflects the cumulative burden of interacting pathological processes rather than the isolated effects of amyloid pathology.

Importantly, co‐pathologies are not merely incidental findings associated with aging but active modifiers of disease expression. Neuropathological studies consistently demonstrate that each additional pathology lowers the threshold of Alzheimer's neuropathologic change required for the development of clinically overt dementia [33, 34]. Conversely, individuals with substantial amyloid pathology but limited co‐existing disease may remain cognitively preserved despite fulfilling biological criteria for Alzheimer's disease. Thus, amyloid burden should be interpreted within the broader pathological context in which it occurs. In many individuals clinically diagnosed with Alzheimer's dementia, cerebrovascular lesions, Lewy body disease, or TDP‐43 pathology may contribute as much as, or even more than, amyloid itself to the emergence and severity of cognitive impairment.

These observations strongly support the contemporary biological framework of Alzheimer's disease. Current NIA‐AA criteria appropriately define Alzheimer's disease according to its core pathological process, as identified by amyloid and tau biomarkers, while recognizing that this biological definition does not capture the full complexity of the clinical phenotype [35, 36].

In accordance, the high prevalence of mixed neuropathology explains why individuals sharing an identical biological Alzheimer's disease profile may experience profoundly different clinical trajectories. Accordingly, the high prevalence of mixed neuropathology provides a biological explanation for the markedly different clinical trajectories observed among individuals sharing similar Alzheimer's disease biomarker profiles.

Viewed through the lens of atherosclerosis, these findings are unsurprising. Just as the clinical consequences of an atherosclerotic plaque depend on the coexistence of systemic inflammation, hypertension, diabetes, thrombogenicity, and other vascular risk factors, the clinical consequences of cerebral amyloid deposition depend on the pathological milieu in which it develops. Amyloid is therefore neither an isolated nor an autonomous determinant of dementia, but one component of a multidimensional biological network that ultimately shapes the transition from pathological aging to clinical Alzheimer's disease.

## 4 The Paradox of Biological Diagnosis

The development of cerebrospinal fluid, blood‐based, and molecular imaging biomarkers has fundamentally reshaped the diagnostic landscape, enabling the identification of Alzheimer's pathology decades before the onset of cognitive symptoms [37]. This paradigm shift has culminated in the recent NIA‐AA criteria, which define Alzheimer's disease according to the presence of its core biological abnormalities, independently of clinical status [36].

Paradoxically, however, the presence of Alzheimer's pathology does not necessarily predict its clinical expression. A substantial proportion of cognitively unimpaired individuals fulfill biological criteria for Alzheimer's disease, many of whom remain clinically stable throughout life. Conversely, patients with apparently similar biomarker profiles often exhibit markedly different rates of cognitive decline, functional impairment, and disease progression [37, 38].

A positive amyloid biomarker accurately identifies Alzheimer's pathological change, just as the detection of an atherosclerotic plaque accurately identifies a chronic vascular disease process. Neither, however, is sufficient to determine when, or even whether, the individual will experience the corresponding clinical syndrome.

Contemporary biological criteria answer a fundamental question with remarkable precision: Does this individual have biological Alzheimer's disease? However, they are not designed to answer an equally important question: What is this individual's likelihood of developing clinical Alzheimer's disease, over what time frame, and through which biological pathways?

As in atherosclerosis, where plaque detection now serves as the starting point for multidimensional risk stratification rather than a diagnostic endpoint [10], we argue that Alzheimer's disease is approaching a similar conceptual transition. The biological diagnosis of Alzheimer's disease should increasingly be regarded as the starting point rather than the endpoint of clinical decision‐making. Once biological Alzheimer's disease has been established, the next challenge is to estimate the probability, timing, and trajectory of clinical conversion by integrating amyloid and tau biomarkers with measures of neurodegeneration, cerebrovascular injury, co‐existing proteinopathies, neuroinflammation, genetic susceptibility, systemic metabolic health, cognitive reserve, and other determinants of resilience or vulnerability.

Importantly, this proposal does not challenge the current biological definition of Alzheimer's disease but builds directly upon it. Rather than asking simply whether Alzheimer's disease is biologically present, future diagnostic frameworks should also seek to determine who will progress, when progression is likely to occur, and why individuals with apparently similar pathological burden experience profoundly different clinical trajectories.

The next evolution of Alzheimer's disease diagnostics lies in moving from biological diagnosis to biological risk stratification. Amyloid and tau biomarkers establish the presence of Alzheimer's pathology; additional biological and clinical modifiers define its likely clinical consequences. This may represent the conceptual framework needed to fully realize precision medicine in Alzheimer's disease.

## 5 Biological Resilience to Amyloid Pathology

One of the most important questions emerging from the biological era of Alzheimer's disease is no longer whether amyloid pathology is present, but why apparently similar pathological burden leads to profoundly different clinical outcomes. Explaining this heterogeneity has become one of the major challenges in Alzheimer's disease research and has shifted attention toward the concept of biological resilience.

Biological resilience should be distinguished from related concepts such as brain reserve and cognitive reserve. Brain reserve refers to structural characteristics, including larger brain volume or greater synaptic density that enable the brain to tolerate greater pathological burden before clinical symptoms emerge [39]. Cognitive reserve reflects the capacity to maintain cognitive performance through more efficient or flexible neural networks, influenced by education, occupational complexity, lifelong cognitive engagement, and other environmental exposures [40]. Biological resilience, in contrast, refers to the intrinsic ability of the brain to limit the pathological consequences of Alzheimer's disease despite the presence of its defining lesions. It encompasses molecular, cellular, vascular, and immunological mechanisms that attenuate the propagation of pathology, preserve neuronal integrity, or enhance adaptive responses to injury [41].

Evidence supporting this concept has accumulated rapidly over the past decade. The resilient PSEN1 E280A mutation carriers described in the Colombian kindred provide perhaps the clearest demonstration that extensive amyloid accumulation can coexist with preserved cognition when downstream pathological processes are restrained [42]. In both the APOE3 Christchurch and RELN‐COLBOS cases, the remarkable delay in symptom onset was associated not with reduced amyloid deposition, but with a profound limitation of neocortical tau spread. These observations suggest that resilience operates by modifying the brain's response to amyloid pathology rather than preventing amyloid accumulation itself [43]. More broadly, longitudinal biomarker studies consistently show that many cognitively unimpaired amyloid‐positive individuals remain clinically stable for years, further supporting the existence of endogenous mechanisms capable of buffering the effects of Alzheimer's pathology.

Resilience is unlikely to depend on a single pathway. Instead, it probably emerges from the interaction of multiple biological systems acting across the course of disease. Experimental and clinical studies increasingly implicate modulation of tau propagation, preservation of synaptic integrity, regulation of innate immune responses, maintenance of blood–brain barrier function, cerebrovascular health, mitochondrial function, proteostasis, and metabolic homeostasis as key determinants of an individual's capacity to tolerate amyloid pathology [44, 45, 46, 47]. Genetic modifiers, including variants in APOE, RELN, and genes involved in microglial biology such as TREM2, further demonstrate that susceptibility and resilience are, at least in part, biologically encoded [48, 49]. At the same time, lifestyle‐related factors, including physical activity, cardiovascular risk control, sleep quality, cognitive stimulation, and social engagement, may enhance resilience by maintaining overall brain health and reducing the impact of downstream pathological cascades. The 2024 Lancet Commission on dementia prevention, intervention, and care provides a particularly informative framework [50]. The Commission identified 14 potentially modifiable risk factors that together account for a substantial proportion of dementia cases worldwide, including lower educational attainment, hearing loss, high low‐density lipoprotein cholesterol, depression, traumatic brain injury, physical inactivity, diabetes, smoking, hypertension, obesity, excessive alcohol consumption, social isolation, air pollution, and untreated vision loss. Traditionally, these factors have been viewed primarily as contributors to dementia risk. However, from the perspective proposed here, they may be more appropriately interpreted as determinants of the brain's resilience, or vulnerability, to Alzheimer's pathology.

Many of these factors converge on biological pathways that are increasingly recognized as critical modifiers of disease progression. Hypertension, diabetes, obesity, dyslipidaemia, smoking, and air pollution promote endothelial dysfunction, blood–brain barrier impairment, cerebrovascular injury, oxidative stress, and chronic systemic inflammation, thereby lowering the threshold at which amyloid pathology translates into neuronal dysfunction. Conversely, higher educational attainment, cognitive stimulation, physical activity, social engagement, and preservation of sensory function contribute to maintaining neural network efficiency, synaptic plasticity, and cognitive reserve, enabling the brain to better tolerate pathological burden. These observations suggest that the clinical consequences of amyloid deposition depend not only on the pathological process itself but also on the cumulative biological environment in which it develops.

Importantly, this interpretation reframes the role of modifiable risk factors within the biological era of Alzheimer's disease. Rather than acting independently of Alzheimer's pathology, these factors may influence the probability that biologically defined Alzheimer's disease progresses to clinical dementia. In other words, they are not simply risk factors for dementia; they are potential modifiers of the brain's response to amyloid pathology. This perspective reconciles two concepts that have often been considered separately: the biological definition of Alzheimer's disease based on amyloid and tau biomarkers, and the substantial reduction in dementia risk achievable through modification of vascular, metabolic, behavioral, and environmental exposures.

This integrated framework closely mirrors the evolution of cardiovascular medicine. In atherosclerosis, the presence of plaque identifies the underlying biological disease, but the occurrence of myocardial infarction or stroke depends on the cumulative influence of systemic cardiovascular risk factors that modulate plaque stability and vascular vulnerability. Similarly, amyloid deposition establishes the biological substrate of Alzheimer's disease, whereas hypertension, diabetes, dyslipidemia, obesity, chronic inflammation, cerebrovascular dysfunction, lifestyle, and cognitive reserve may collectively determine whether this pathology remains clinically silent or progresses to dementia. From this perspective, the 14 modifiable factors identified by the Lancet Commission can be viewed not only as targets for dementia prevention, but also as determinants of biological resilience that influence the clinical expression of Alzheimer's pathology.

This conceptual shift has important implications for both biomarker interpretation and therapeutic development. Current biomarkers accurately identify the presence of Alzheimer's pathology but provide limited information regarding an individual's capacity to tolerate that pathology. Future risk assessment should therefore integrate measures of pathological burden with biomarkers reflecting vascular integrity, neuroinflammation, synaptic function, metabolic health, and other biological processes associated with resilience. Such an approach would enable clinicians not only to diagnose biological Alzheimer's disease but also to estimate the likelihood and pace of clinical progression.

Ultimately, biological resilience provides the missing link between pathological burden and clinical phenotype. It may explain why individuals with apparently comparable amyloid deposition follow markedly different trajectories, why anti‐amyloid therapies produce heterogeneous clinical responses, and why modifying vascular and lifestyle‐related factors remains beneficial even after Alzheimer's pathology has developed. Rather than viewing resilience as an exception to the disease process, it should be regarded as a fundamental biological determinant of disease expression. If amyloid defines the biological identity of Alzheimer's disease, resilience may ultimately define its clinical destiny.

## 6 Toward a Multidimensional Model of Alzheimer's Disease

The evidence discussed above converges toward a multidimensional model of Alzheimer's disease in which clinical progression reflects the balance between mechanisms that amplify pathological injury and those that preserve brain function. Tau propagation, neurodegeneration, cerebrovascular dysfunction, neuroinflammation, synaptic integrity, systemic metabolic health, co‐existing proteinopathies, genetic background, and biological resilience collectively contribute to the heterogeneous trajectories observed across the Alzheimer's disease continuum.

**FIGURE 2:**
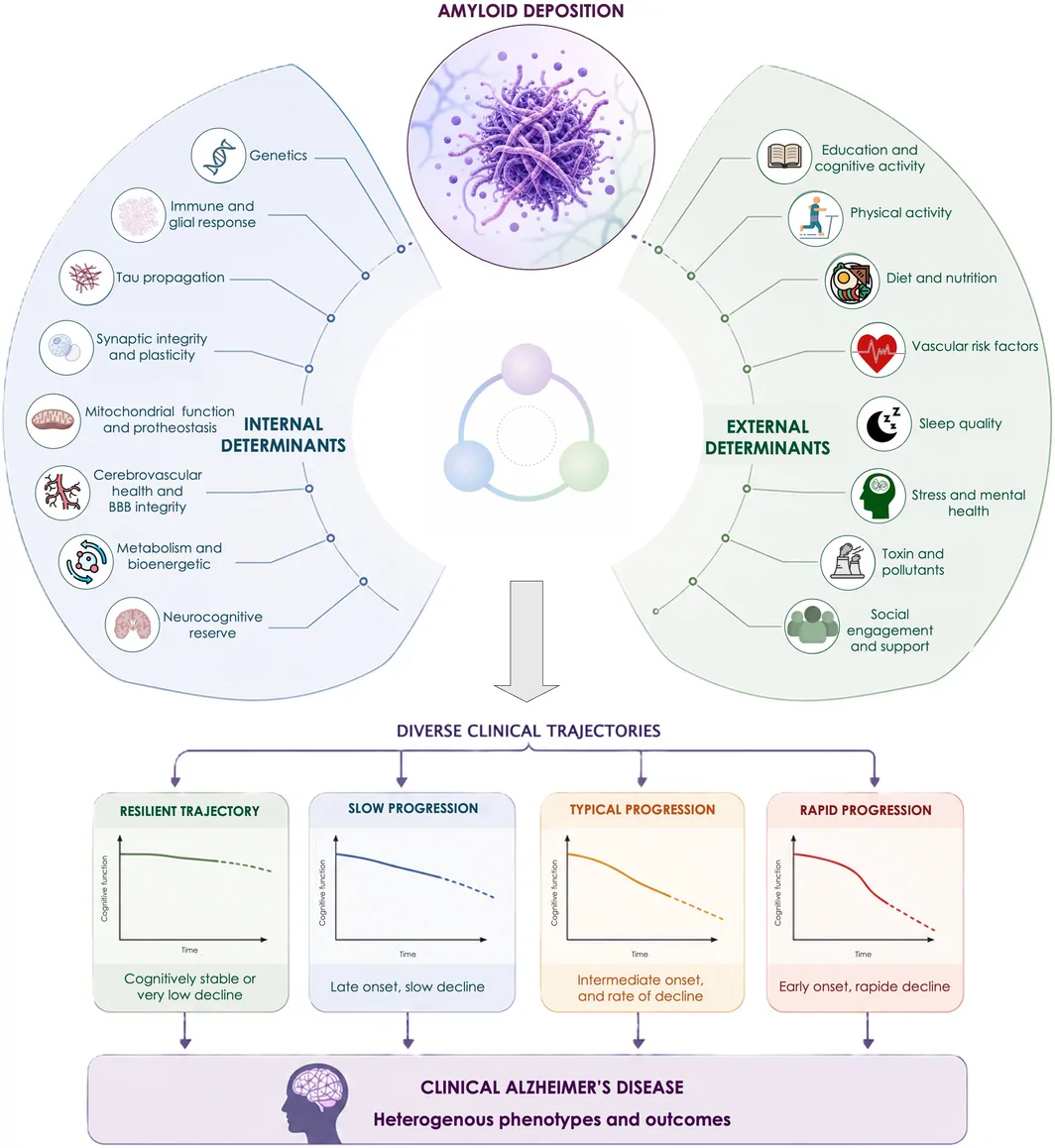
From biological Alzheimer's disease to clinical Alzheimer's disease: a multidimensional framework of disease expression. The clinical expression of biological Alzheimer's disease results from the interplay between internal determinants of resilience and vulnerability and external, potentially modifiable factors, leading to heterogeneous disease trajectories.

Within this framework, amyloid should no longer be viewed as an isolated determinant of disease progression but as the initiating component of a dynamic biological network (Figure 2). The clinical phenotype emerges not from amyloid burden alone, but from the balance between processes that amplify pathology and those that preserve brain function despite ongoing pathological change. As illustrated in Figure 2, the transition from biological Alzheimer's disease to clinically manifest disease may be conceptualized as the result of a dynamic balance between internal and external determinants of resilience and vulnerability. Internal determinants include genetic background, regulation of tau propagation, innate immune and glial responses, synaptic integrity, mitochondrial function, cerebrovascular health, metabolic homeostasis, and neurocognitive reserve. These interact with external and potentially modifiable determinants, including education, cognitive stimulation, physical activity, nutrition, cardiovascular risk factor control, sleep quality, psychosocial well‐being, environmental exposures, and social engagement. Their cumulative interaction may contribute to markedly different clinical trajectories, ranging from prolonged cognitive resilience despite Alzheimer's pathology to more rapid progression toward cognitive impairment and dementia.

The NIA‐AA biological criteria, the evidence from anti‐amyloid therapies, the high prevalence of mixed neuropathology, biological resilience, and the Lancet Commission's modifiable risk factors are complementary dimensions of the same disease process: amyloid and tau biomarkers define the biological identity of Alzheimer's disease; co‐pathologies, vascular and metabolic health, neuroimmune responses, genetics, and resilience determine its clinical expression.

## 7 Conclusion

The history of cardiovascular medicine demonstrates that identifying a pathological lesion is only the first step toward understanding disease. Clinical events ultimately emerge from the interaction between the lesion and the biological context in which it develops. We argue that Alzheimer's disease is undergoing a similar conceptual evolution. Amyloid remains the defining pathological substrate of the disease, but its clinical consequences are determined by a multidimensional network of biological, genetic, vascular, metabolic, environmental, and resilience‐related factors. Recognizing this distinction places amyloid within a broader framework that better explains the heterogeneity of disease trajectories and therapeutic responses. The next frontier in Alzheimer's disease research is therefore not to move beyond amyloid, but to move beyond an amyloid‐centric interpretation of the disease. Integrating pathological burden with the biological determinants of resilience and vulnerability may provide the foundation for truly personalized prediction, prevention, and treatment.

## Author Contributions

L.A. and M.C. jointly conceived the manuscript and its central conceptual framework. L.A. performed the literature review and drafted the manuscript. M.C. critically revised the manuscript, contributed to the interpretation of the literature, and supervised the work. Both authors contributed to the development of the final version and approved the submitted manuscript.

## Funding

The authors have nothing to report.

## Conflicts of Interest

The authors declare no conflicts of interest.

## Data Availability

The authors have nothing to report.
